# The MadQCI Cloud Scenario: Quantum as a Service

**DOI:** 10.3390/e28030283

**Published:** 2026-03-02

**Authors:** Jaime S. Buruaga, Alberto Sebastián-Lombraña, Ruben B. Méndez, Rafael J. Vicente, Juan P. Brito, Laura Ortiz, Vicente Martin

**Affiliations:** 1 Center for Computational Simulation, Universidad Politécnica de Madrid, 28660 Madrid, Spain; aj.sebastian@upm.es (A.S.-L.); ruben.bmendez@upm.es (R.B.M.); rafaelj.vicente@upm.es (R.J.V.); juanpedro.brito@upm.es (J.P.B.); laura.ortiz@upm.es (L.O.); vicente.martin@upm.es (V.M.); 2 DLSIIS, ETSI Informáticos, Universidad Politécnica de Madrid, 28660 Madrid, Spain; 3 DATSI, ETSI Informáticos, Universidad Politécnica de Madrid, 28660 Madrid, Spain

**Keywords:** quantum technologies, quantum as a service, quantum key distribution, post-quantum cryptography, quantum random number generator

## Abstract

Within the Madrid Quantum Communication Infrastructure (MadQCI), a cloud-like, quantum-enabled network scenario has been commissioned to promote the growth of the quantum technology scientific community. This scenario is designed to provide both quantum communication primitives and quantum-enabled services to potential end users. This work focuses on exposing these quantum services in a user-friendly manner by abstracting the underlying technical complexity, letting end users operate without prior knowledge of implementation details. To this end, multiple quantum services—the SD-QKD software stack, QRNG, Quantum-Safe TLS, and Quantum-Safe IPsec as a Service—are offered following the cloud “anything as a service” (XaaS) model. The delivery of quantum-enabled services is therefore researched using an applied and transferable cloud-based paradigm.

## 1. Introduction

The scalable and adaptable delivery of quantum-enabled services is a fundamental topic for the wide adoption of quantum communications. In the MadQCI, a distributed, quantum-enabled data centre has been deployed to support third-party R&D activities in a cloud way.

The Madrid Quantum Communications Infrastructure ecosystem, namely, the MadQCI ecosystem, is a metropolitan testbed focused on the deployment and integration of quantum communication technologies in current telecommunication infrastructures [1]. This mainly includes quantum key distribution (QKD) and quantum random number generation (QRNG) systems, allowing several quantum cryptography techniques [2,3]. This quantum network is therefore aligned with the EuroQCI [4], a pan-European initiative to deploy large, quantum-enabled networks devoted to secure communications. Furthermore, to research how to adapt and scale quantum communication to real-world applications, this iteration of the MadQCI ecosystem was designed with several scenarios [5].

The primary objective of this work is to report on the MadQCI “Demonstration” scenario [6], which was designed to open some capabilities of the quantum network to third parties. This makes collaboration on research and development, as well as the promotion of the advancement of quantum communication technologies, possible while addressing the issue of delivering quantum-enabled services to realistic end users. These capabilities include fundamental quantum network functions, such as QKD and QRNG, as well as abstracted ones that mimic real-world, quantum-enabled services.

To fulfil this aim, a distributed data centre-like infrastructure was deployed along three nodes of the network using both classical technologies—e.g., three IT systems each connected through a pair of optical fibres—and quantum network systems. To enhance the service delivery, quantum capabilities have been made available using the “anything as a service” (XaaS) cloud approach. In this way, the adaptability and scalability of quantum technologies are specifically researched while taking advantage of the capabilities offered by cloud technologies.

This paper shows both the infrastructure designed and deployed and the quantum-enabled services made available in the scenario, and the delivery of these services using a cloud approach is discussed. First, we present some fundamentals of cloud computing and quantum networking (Section 2), followed by a detailed description of the proposed scenario (Section 3). We then discuss the services proposed under the “quantum as a service” model (Section 4). Finally, Section 5 presents the conclusions and outlines the potential directions for future research.

### Related Works

This scenario was proposed to continue certain collaborations with third parties that were already underway in the Madrid quantum network laboratories using virtual machines as a gateway to the ETSI GS QKD 004 [7] and ETSI GS QKD 014 [8] access points of the quantum network. It was reported as an effort of the OpenQKD project [9]. The goal of this work was to scale up that approach, exploiting the benefits of using more complex frameworks and expanding the range of services provided. However, it also finds inspiration in other data centre-like infrastructures that offer quantum-based cloud services. A good example of this is quantum computing as a service, delivered by large companies such as Amazon Braket, Azure Quantum, Google Quantum AI, and IBM Q [10]. They provide quantum computation services such as interfaces to program quantum circuits and backends to execute them on real quantum computers. These services are based on code stacks such as Qiskit [11], which also perform tasks for the end user, such as automatically generating circuits for the most well-known algorithms, encoding certain high-level problems in those algorithms, or low-level tasks such as cross-compiling to the actual qubit architectures of quantum computers. This enables end users to execute quantum-enabled programs without being experts on quantum computing. Analogously, this work is driven by the ability to expose quantum-based functionalities in a more abstract and user-friendly way.

Regarding quantum communication technologies, to the best of the authors’ knowledge, there have been only isolated and transversal efforts. This includes the provision of random numbers from QRNG through application programming interfaces [12,13], often referred to as entropy as a service (EaaS); the delivery of cloud-native simulation of quantum network architectures, such as Quditto [14]; and research on integration of certain quantum network functions into the cloud, such as monitoring QKD systems from the cloud [15].

This work reports on an effort to (1) provision an entire data centre-like infrastructure from scratch using suitable quantum technologies while (2) researching cloud-based delivery of quantum-enabled services (3) prepared for real third-party end users.

## 2. The Cloud for Quantum Networking: Fundamentals

This section details the fundamental concepts of the cloud computing approach adopted in this work, as well as those of the quantum communication infrastructures and quantum network systems in which the cloud approach is applied.

### 2.1. Cloud Computing and Service Delivery

Cloud computing is a model that enables ubiquitous, user-friendly, on-demand access to a shared pool of computing resources—e.g., applications and storage—that can be rapidly provisioned and released [16,17,18]. This paradigm is based on the idea of offering infrastructure and services in an abstract, remote, and elastic manner, i.e., adapting to user demand. Essential characteristics of this type of service provision include being pay-per-use, on-demand, and self-service, abstracted from the infrastructure that supports them. The ISO/IEC 17788 standard expands this vision by incorporating aspects such as multi-tenancy and interoperability [18]. Table 1 shows an analysis of the proposed scenario according to the definition of the cloud in [16].

**Figure 1:**
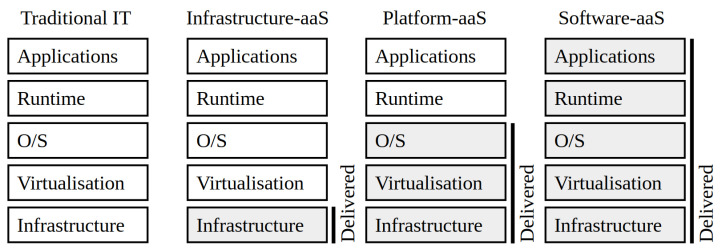
The usual abstraction levels used in cloud computing. Depending on the capabilities made available to the user, it is possible to deliver infrastructure as a service (IaaS), platform as a service (PaaS), software as a service (SaaS), and function as a service (FaaS). This work researches how to adapt this framework to deliver quantum communication services.

In the field of cloud computing, different approaches are used depending on the systems made available to the end user and how they are abstracted. That is the case in the delivery of infrastructure as a service (IaaS), platform as a service (PaaS), software as a service (SaaS), and function as a service (FaaS), for example. Figure 1 illustrates this. This work focuses on researching these possible levels for quantum communication services using the definition of anything as a service.

### 2.2. Quantum Network Systems for Data Centres

Quantum communication systems are still being researched and developed, and there is still a long way to go before there is a “quantum internet” capable of connecting quantum computers [19].

However, several quantum-based systems are currently available on the market. Quantum communication testbeds such as the MadQCI ecosystem demonstrate that these systems are prepared for commercial exploitation, although several issues remain unsolved—e.g., widely adopted standardisation, the coexistence of classical and quantum systems, and deficiencies in the level of service, as addressed in this work.

#### 2.2.1. QKD Systems

The majority of these are quantum key distribution systems, which enable the delivery of symmetric cryptographic keys to two remote systems [2,3]. To do this, the principles of quantum mechanics are leveraged, and, although no practical implementation is free from security risks, it offers unconditional security in theory [20]. This technique will be essential for protecting information when a cryptographically relevant quantum computer exists and breaks other classical key agreement methods [21].

All QKD systems have similarities, although there are several types and even distinctive applications and business models. All of them function as black boxes that, in their fibre optic-based variants, can be integrated into optical networks with standard passive multiplexing optics, i.e., dense wavelength division multiplexing (DWDM) and coarse wavelength division multiplexing (CWDM) equipment. General methods for the coexistence of quantum and classical equipment are still under investigation, although the fundamental means are known [2].

**Figure 2:**
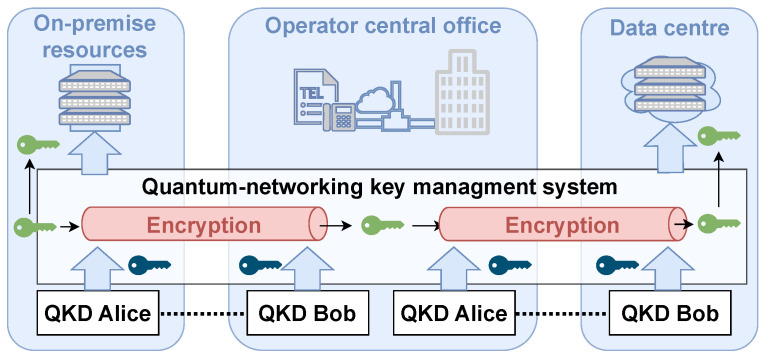
Several premises protected with QKD. Quantum key distribution involves not only growing key material using quantum methods, but also deploying quantum networks that overcome its range limitations. To this end, QKD systems are concatenated in multiple fibre spans between premises, which must be trusted nodes, and session key material is encrypted and decrypted hop by hop. This key material is delivered to end users, typically from a QRNG, rather than that generated in QKD systems, which is solely used for the key forwarding, i.e., encrypting the key material delivered. Several key management and network management quantum network functions make these operations possible [2].

All of them typically deliver the quantum-generated key using standard interfaces such as the ETSI GS QKD 004, ETSI GS QKD 014, and SKIP [22]. This enables the integration of these systems with any current cryptographic application, such as those described in Section 2.3. Furthermore, it enables a more QKD-specific task that is not included in the usual key management system (KMS) procedures [23]: key forwarding. Indeed, QKD links have range limitations, so delivery of long-distance key material is currently needed to transport the key material. To do so, the key material delivered to the end user is extracted on one side from an entropy source—e.g., a QRNG system—and then ciphered in a hop-by-hop basis to the remote side. The Vernam cipher [24] is used to preserve the ITS feature of QKD, as well as other cryptographic primitives to enforce integrity and authentication. Figure 2 shows this approach, which involves an area of quantum networking dedicated to investigating the distribution of key material between remote locations using QKD [2]. It covers which network-aware techniques are best suited to controlling quantum network systems—e.g., the SD-QKD approach of the ETSI GS QKD 015; which key management and forwarding techniques enable optimal service performance—e.g., aggregating multiple multi-path key streams to serve a single pair of end users; and how to integrate all of this into existing infrastructures.

**Figure 3:**
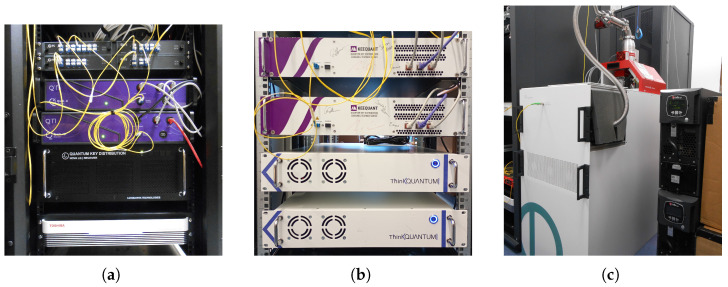
Several examples of QKD systems deployed or being tested in the Madrid quantum network. The technology procured for the MadQCI ecosystem was selected to fit each application scenario, not the other way around. In this way, this technology enables other technologies specific to each application, allowing for the generation of applied and transferable knowledge, and, since each piece of technology is best suited to different uses, the MadQCI ecosystem is a showcase for the entire European quantum ecosystem, with practically all of its manufacturers represented. (**a**) Several QKD systems in the Rectorado node of the MadQCI. The Toshiba at the bottom is the actual Toshiba deployed for this quantum network scenario. QKD operation is supported by other quantum network systems such as optical and Ethernet equipment and IT systems. (**b**) A KEEQuant CV-QKD system on top and a ThinkQuantum DV-QKD below being tested at the laboratory. Several types of QKD exist, with different performance and network integration features, so an assessment is needed to choose the most suitable quantum system for each application. (**c**) A ZeroThird entanglement-based QKD system is commissioned with a vacuum pump; the UPS system on the right prevents vacuum losses in the event of a power failure. Its integration was more challenging, but the distribution of quantum entanglement will enable the “quantum internet”.

QKD technology is currently establishing itself in the market with multiple business models, which conditions its integration. Figure 3 shows several QKD systems of the MadQCI ecosystem.

Many manufacturers choose point-to-point solutions, such as HEQA, ID Quantique, QTI, ThinkQuantum, or Toshiba in discrete-variable QKD and KEEQuant or LuxQuanta in continuous-variable QKD. The foundations of the former ones are better known and show better performance over long distances, although the latter have more potential for scalability and classical–quantum coexistence. Furthermore, each brand exhibits its own distinctive features. Different designs and configurations, richer sets of KMS functionalities, research and external detection features, interoperability with free-space equipment, and tunability of the optical channels are some examples of that variety.

Other vendors take advantage of the new QKD techniques to offer alternative competitive advantages, such as Q*Bird, which uses systems based on measurement device-independent QKD [25] to offer a configuration based on a central hub with multiple emitters arranged in a star configuration. ZeroThird QKD systems, formerly Quantum-Industries and shown in Figure fig:systemsc, are based on quantum-entangled photons and high-efficiency detection, which resembles the fundamental techniques of the future quantum internet.

A potential QKD breakthrough will come when miniaturisation in on-chip photonics enables other form factors, such as small form-factor pluggable (SFP) integrations, and, particularly, lower equipment costs. In addition, the leap to the space segment would enable worldwide quantum connectivity [19].

#### 2.2.2. QRNG Systems

Quantum random number generation systems can be used both in quantum cryptography—e.g., to generate quantum-distributed keys in multi-hop quantum networks—and in any application that benefits from high-quality entropy sources, such as Monte Carlo optimisation and gambling. Therefore, these devices offer significant potential in cloud-based environments, where entropy can be consumed by multiple services and applications on demand.

QRNG systems are available in several formats. Both standalone appliances and PCIe-based QRNG systems are available from vendors such as ID Quantique, Quantum Dice, QuintessenceLabs, Quside, and Thinkquantum. These solutions are capable of delivering several hundreds of Mb/s of quantum-generated random numbers, although the Quantum-dice and QuintessenceLabs options may require a licence. QRNG appliances typically provide an entropy-as-a-service approach through a REST API, while PCIe-based solutions are often delivered with software development kits that allow direct integration and exploitation of their capabilities.

#### 2.2.3. Post-Quantum Cryptography

Post-quantum cryptography (PQC) [26] is a cost-effective and standardised alternative to QKD that is being actively promoted by leading institutions in the field of information security. PQC schemes provide resistance against known threats from quantum computing, although its intrinsic risks are unknown at this time [27].

In the context of data centres, PQC represents a suitable solution for managing information security within controlled premises. PQC provides bit-strength security equivalent to that required by widely used cryptographic applications such as AES or MACs. Moreover, it is currently being integrated into widespread technologies such as OpenSSL [28] and TLS [29]. Therefore, assuming that qualified physical security is in place on the premises, it is reasonable to conclude that PQC is the most cost-effective, scalable, and adaptable solution for securing intra-data centre communications. This includes security between different IT systems, but also between the different virtualised resources available to users within the same premises.

### 2.3. Quantum-Enabled Services

Quantum cryptography delivers key material that may be consumed by any usual cryptographic application that requires a key agreement primitive to work. In this regard, research is ongoing on how to integrate QKD with these applications, often hybridised with other key material—e.g., PQC and pre-shared key material. Thus, a quantum-enabled service is any application that consumes quantum-distributed keys to deliver functionality to end users.

Several applications are particularly relevant for the cloud environment. For example, virtual private networks (VPNs) enable remote access to private networks through secure tunnels over the internet, exposing resources as if the end users were on the same private network while protecting data in transit. In practice, VPNs are commonly implemented using IPsec- (e.g., ESP with IKEv2) or TLS-based solutions (e.g., OpenVPN). IPsec can provide confidentiality, integrity, and data origin authentication for IP traffic (often including anti-replay protection) and is widely used for both site-to-site and remote-access deployments, remaining transparent to the protected applications. TLS (Transport Layer Security) is widely used by application protocols (e.g., HTTPS) to secure client–server communication for websites and APIs.

This work uses quantum-enabled versions of these primitives to research how to deliver quantum-enabled services to the end user in a cloud style.

### 2.4. Quantum-Enabled Cloud Model

Finally, it is worth outlining a model that brings quantum communication technologies closer to the provision of services such as cloud computing.

This work uses an anything-as-a-service approach, in which the level of abstraction of the more traditional cloud services, IaaS, PaaS, SaaS, etc., is not so relevant. In this sense, abstraction may be understood as a continuum that ranges from quantum network infrastructure to the most abstract cryptographic services. This flexibility is important given how different quantum-enabled services are from those typically provided in cloud environments. However, the discussion of which service model applies to each type of service in a quantum communications network remains valuable, as it provides an appropriate conceptual framework for devising them. A valid approach is illustrated in Table 2.

Furthermore, other axes may emerge when discussing this topic, such as delivering general-purpose or specific-purpose services, e.g., delivering quantum-generated random numbers using an agnostic API or using specific formats for mathematical libraries that perform Monte Carlo methods and for cryptographic standard interfaces such as PKCS#11. The service delivery performance and experience of the end user will be very different in each of these cases.

Finally, it is important to note that the same technique or system can enable multiple service models, which is why an XaaS approach is more appropriate. For example, the ETSI GS QKD 004 standard API can enable the virtualisation of several QKD systems by distributing the key generated by a single device, corresponding to an IaaS. However, it also provides a set of methods for composing different quantum services that can be delivered as PaaS since calls to this API allow configuration of quality of service-oriented key delivery parameters: minimum and maximum key rates per second, delivery jitter, key expiration, packet size, etc. An end user could use this API to design the delivery of AES-128 keys every minute, or to obtain a continuous key seed for a distributed Monte Carlo method. Finally, SaaS and FaaS models could be delivered by exposing this API through a textual or graphical interface to end users.

## 3. Quantum Network Infrastructure: Implementation and Findings

This section elaborates on the quantum network infrastructure that supports this research, as well as some specific results and conclusions interesting for quantum-enabled cloud deployments.

### 3.1. MadQCI Ecosystem

**Figure 4:**
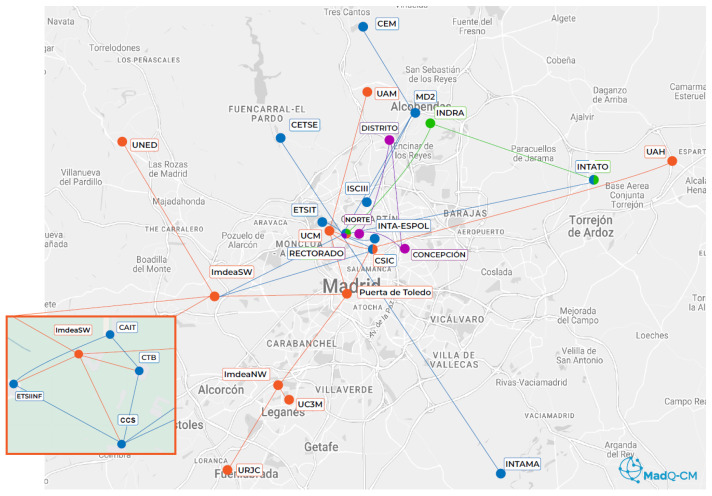
The MadQCI ecosystem consists of the interconnection of a simpler QCI. In orange: the MadQCI network that connects all public universities in Madrid, operated by REDIMadrid (IMDEA Software). In blue: the interconnections that UPM provides with other institutional nodes, such as other research and high-security centres. The two industrial stakeholders are in purple (Telefónica Innovación Digital) and green (Indra).

Figure 4 shows the MadQCI ecosystem, based on the interconnection of several quantum communication infrastructures (QCI), including the nodes managed by Universidad Politécnica de Madrid (UPM) and REDIMadrid (RM, operated by IMDEA Software), the regional research and education network, and the nodes operated by Indra and Telefónica Innovación Digital (TInD), devoted to industrial and commercial applications.

UPM connects many highly relevant actors, such as the Centro Español de Metrología (CEM), which has an optical clock; the Centro Tecnológico de Seguridad (CETSE), a police institution; the Consejo Superior de Investigaciones Científicas (CSIC); and several branches of the Instituto Nacional de Técnica Aeroespacial (INTA), all related to military facilities.

The MadQCI ecosystem is designed with scenarios devoted to specific applications, which respond to this heterogeneous set of locations and stakeholders [5]. Each scenario features classical and quantum technology aligned with the usual requirements of the specific quantum networking or niche applications, although the entire ecosystem has end-to-end quantum connectivity.

These scenarios in the MadQCI ecosystem range from basic quantum networking applications, such as quantum–classical coexistence and switching, to more transferable or institutional ones, such as high-security applications or the cloud implementation reported in this work. In some locations, up to five scenarios coexist in a single location, although only the rack cabinets and management network are shared; each scenario uses its own fibre optic pair and its own specific set of technology, both classical and quantum. Inter-domain interoperability at the quantum network infrastructure level has also been aimed at testing multiple solutions.

In general, the quantum network infrastructure has been designed, procured, and installed to trial quantum technology and quantum-enabled services over the next decade. Public procurement of quantum technology benefitted from the multi-scenario approach, as it enabled the acquisition of a diverse set of QKD and QRNG systems with different characteristics. Thus, almost the entire European quantum communication ecosystem is represented: HEQA, ID Quantique, KEEQuant, LuxQuanta, QTI, Quantum-Industries, Q*Bird, ThinkQuantum, and Toshiba QKD vendors, as well as QRNG of ID Quantique and Quside. This heterogeneity promotes vendor-independent research and, therefore, prepares it for technological transfer to industry.

### 3.2. Scenario-Specific Infrastructure

The scenarios in the MadQCI ecosystem have been designed so that the technology adapts to the specific application and not the other way around. This includes both classical and quantum technology and is the most effective way to generate applied and transferable knowledge.

**Figure 5:**
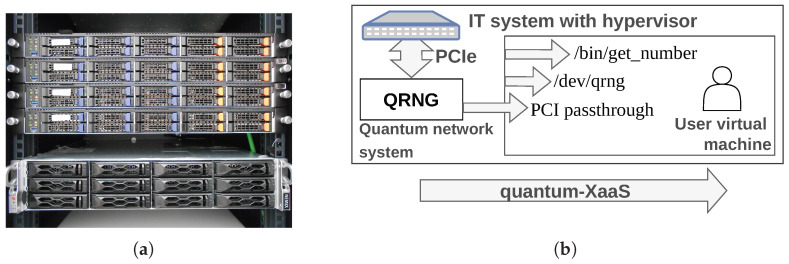
The complexity of this scenario lies in the software and how services are provided to end users of the cloud. (**a**) The IT systems of the cloud-type scenario when they were being commissioned—3 nodal IT systems, an additional one for gateway tasks, and a network-attached storage IT system. Their quantum and classical connectivity is point-to-point, and the QRNG and hardware security module functionalities are integrated as PCIe cards. (**b**) Approach for exposing q-XaaS inside the IT systems. A single quantum resource, such as QRNG, may be abstracted in several ways to deliver its services to a multitude of end users. In the picture, up to three possible sources of entropy are exposed to the end user: (i) the QRNG itself, (ii) the usual Linux-based RNG descriptor /dev/random, and (iii) software or a function installed in the path /bin/get_rn. Each end user may choose a different approach depending on their expertise level and exposed capabilities. Furthermore, as stated in Table 2, several IaaS, PaaS, SaaS, FaaS, etc., quantum-enabled services can be delivered to the end user.

Figure 5 shows the IT systems that were procured to deploy the distributed cloud-type scenario. These consist of three servers with high virtualisation capabilities, as well as one dedicated to gateway tasks and a NAS—the latter shared with other scenarios on the network. Each of the three main IT systems has 64 processors at 2.45 GHz, as well as 256 GB of RAM. These capacities were sized to support up to 10 complex users—with more dedicated resources—and 20 simpler users, all of them present simultaneously on the three machines. Finally, high network connectivity capabilities were procured: a 25 GbE/s connection for out-of-band management and quantum-related networks and up to 100 GbE/s and Infiniband EDR100 for point-to-point application connectivity.

Figure 5 also depicts the approach to quantum-XaaS tested in each IT system. This means that the complexity of this scenario does not lie in its infrastructure, but in what it houses. Exposing quantum-enabled services to the user is not straightforward due to both the inherent complexity of designing each service delivered and the technical complexity of the proposed solution. In this way, quantum and classical technology in this scenario is in the service of the cloud-type application and not the other way around, leveraging applied research in this field as a cutting-edge enabling technology.

**Figure 6:**
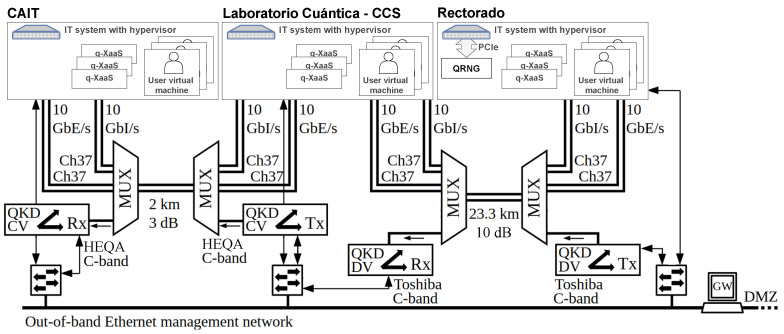
Infrastructure elements supporting the quantum-enabled distributed data centre for open R&D presented in this work. These include both quantum technology for delivering q-XaaS—i.e., QKD and QRNG systems—and classical equipment for supporting its operations. The out-of-band management network is shared with other MadQCI colocated scenarios: UPM-Trunk, UPM-beyond-QKD, UPM-high-security, and UPM-switching, for example.

The deployed quantum network infrastructure mimics the one that could be found when connecting several data centres that support cloud-type operations. Indeed, unlike other applications where network complexity can be challenging, connectivity between data centres can be point-to-point and supported with only a few optical channels (up to 800 GbE/s optical transceivers are commercially available). Thus, Figure 6 shows how the connectivity of the three IT systems is point-to-point, multiplexed with two QKD systems that enable secure key exchange. The limited number of coexisting classical channels allows for integration with the most flexible optical infrastructure since multiple vendors support it. Note that, in this case, intra-data centre connectivity has not been addressed, which is a challenge in itself but does not necessarily need to be protected by quantum cryptography if the premises are sufficiently protected and act as trusted nodes.

The installed QKD technology was chosen to suit this infrastructure. First, in the smallest fibre span, a QKD system in the O band provided by HEQA was chosen for integration. This system is, to the authors’ knowledge, the cheapest on the market, but it allows for reasonable performance; its installation in a fibre span with no more than 3 dB loss was ideal for testing optical coexistence with DWDM channels in the C band and currently performs in coexistence with a 10 GbE/s DWDM C46 optical channel. In the longer span of 23 km and approx. 10 dB of optical losses, a C-band Toshiba QKD system is being tested for two reasons: First, it is the point-to-point discrete-variable QKD system that performs better in terms of key rate, which may be needed to deliver keys in cloud environments to several end users requesting key material. Secondly, its design includes multiplexing features, which would ease the integration in point-to-point links such as the typical ones for connecting data centres, although this feature has yet to be tested in the MadQCI.

**Figure 7:**
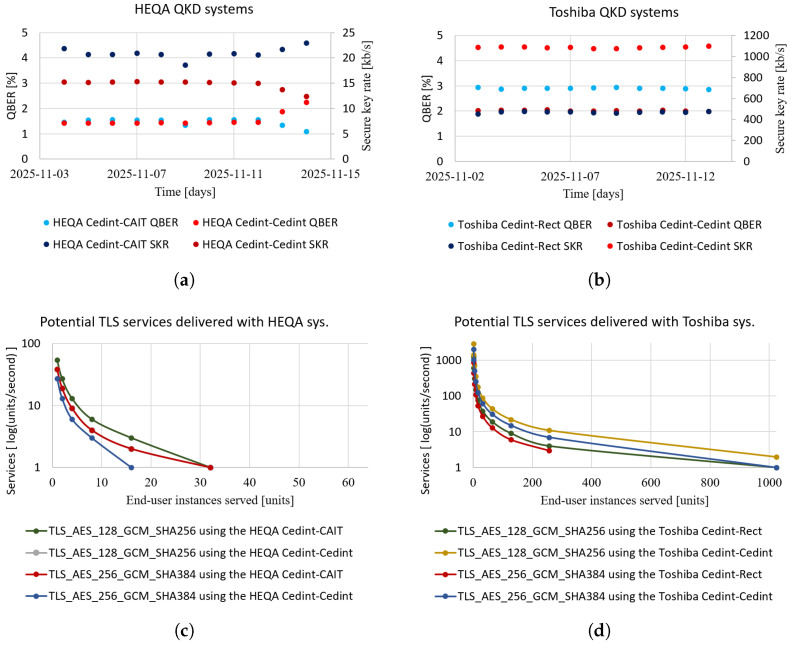
This figure illustrates the potential of quantum network technologies to provide services in cloud environments. (**a**,**b**) show the QBER and secure key rate (SKR) delivered by four QKD systems: the HEQA and Toshiba systems deployed and the other HEQA and Toshiba systems being tested in a back-to-back configuration on the same premises. The differences in the overall performance between the two brands, due to its proprietary features, are notable. Secondly, there is a clear correlation between the performance of each pair of systems, showing that the environmental conditions of the data centre may have more impact than the deployed optical fibre. (**c**,**d**) show the potential usage of these systems. Using the mean values of the key rates, the potential performance and scalability of the QKD systems are shown for two of the most-used TLS suites, i.e., using 384- and 640-bit symmetric keys in each instance. It is assumed that there is no rekeying. (**a**) Measured secure key rate and QBER of two HEQA systems, one deployed between CCS and CAIT nodes, in coexistence with a 10 GbE/s DWDM channel, and a second one being tested in the CCS node in a back-to-back configuration. (**b**) Measured secure key rate and QBER of two Toshiba systems, one deployed between CCS and Rectorado nodes and a second one being tested in a CCS node in a back-to-back configuration. (**c**) Potential TLS services per second delivered using both the deployed and back-to-back QKD HEQA systems of (**a**), considering both the TLS suite and the number of end user instances served. (**d**) Potential TLS services per second delivered using both the deployed and back-to-back QKD Toshiba systems of (**b**), considering both the TLS suite and the number of end user instances served.

Figure 7 shows the performance of these two systems, as well as the expected performance when delivering quantum-enabled services. Regarding the former, Figure 7a,b show performance metrics, both for the two systems deployed and for two other systems from the same vendors that were being evaluated back-to-back in the same room. Figure 7c,d show a possible quantum-enabled service that could be provided using these systems, in this case, TLS services that use 384- and 640-bit symmetric keys in each instance.

Regarding the cost, the HEQA QKD systems were procured in an open and public procedure for €118,000, while the Toshiba ones cost €187,000. Table 3 shows the CAPEX of these service deliveries assuming a 5-year depreciation period for the QKD systems—usually, telecommunications technology maintenance is very costly after 5 years, so deployment costs are often calculated for that time. Finally, regarding the QRNG, different prices arise depending on the type of system, ranging from less than €5000 for the PCIe ones to more than €15,000 for the appliance entropy-as-a-service ones.

## 4. Quantum as a Service: Enabled Implementation and Findings

**Figure 8:**
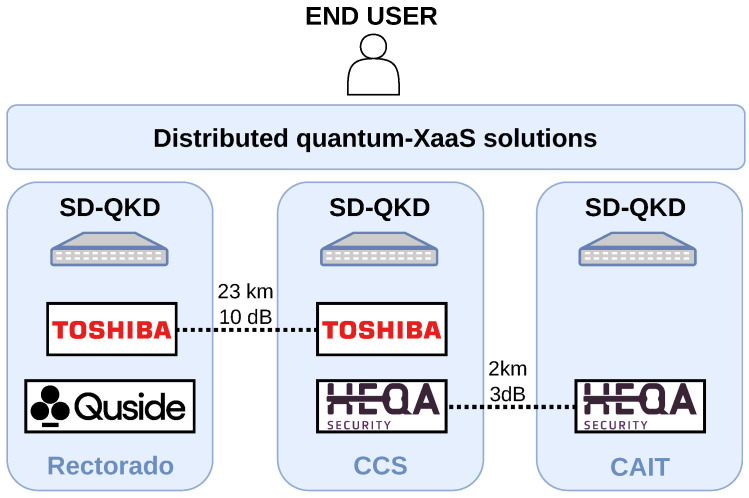
High-level MadQCI cloud scenario overview. The scenario is composed of three SD-QKD servers, two Toshiba QKD systems, two HEQA systems, and a QuSide Garnet™.

This section describes the implementation of a set of quantum-enabled services that can enable the delivery of cloud-style services to end users, as Figure 8 depicts, as well as the approach used to provide them. These quantum services were selected given the technologies developed, deployed, and tested in the MadQCI network.

Also, it is important to note that a trust network approach is adopted for exposing all the services, i.e., it is assumed that qualified security is not needed in the intra-data centre premises. HTTPS requests are performed without TLS verification, as both the emitter and receiver reside within the internal network. Authentication and authorisation are enforced at the gateway of the internal network; once inside, all requests are assumed to come from authenticated and authorised entities.

### 4.1. SD-QKD Stack

The UPM domain of the Madrid quantum network uses a software-defined networking approach to quantum communications (SD-QKD) to enable the end-to-end delivery of key material [1]. This software stack is based on the ETSI GS QKD standards so that services can be delivered exposing its capabilities to end users.

This software is capable of managing the key material grown by the QKD systems at any network node to deliver end-to-end quantum-distributed keys. It exposes all available ETSI GS QKD interfaces for delivering the quantum-distributed keys to end users—i.e., ETSI GS QKD 004 [30] and ETSI GS QKD 014 [31], as detailed in the following sections—while making use of the workflows defined in ETSI GS QKD 015 [32] for the management and operation of the quantum network programmable resources.

This software stack is complex to operate and deploy, so, to enable building deliverable services in a cloud way, it was necessary to expose the full set of operations for this software in a user-friendly manner that does not need any know-how. Thus, a REST API was created to expose this functionality, so that both the end user and the cloud administrator only need to make a GET request to the local node against the desired end node, following the legend on Table 4. See Listing 1 for an example.

Example for retrieving a QKD key from CAIT to Rectorado, with key forwarding in CCS, shown in Listing 1.

**Listing 1.** CURL HTTP GET request to retrieve a SD-QKD key from CAIT to Rectorado.

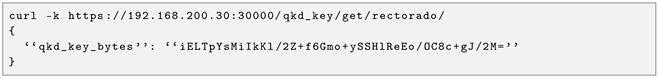



As a result of this request, a 32-byte quantum-distributed key is returned in Base64 format, as shown in Listing 1.

This approach has two fundamental advantages: First, the key material and network topology are managed internally by the software stack, so the end user can choose to extract the key from each of the two hops (CAIT-CCS and CCS-Rectorado) and perform key forwarding or directly extract the key from the CAIT and Rectorado as if there were an end-to-end QKD system. Second, services can be delivered to a multitude of users with only two QKD systems and in a packetised, on-demand, and chargeable way, ultimately enabling the delivery of quantum-enabled services with a cloud model.

### 4.2. SD-QKD Stack: ETSI GS QKD 014

The SD-QKD software stack can expose the ETSI GS QKD 014 interface for those end users who want to integrate the main European standard to retrieve keys from QKD devices in their developments. Note that the SAEs correspond to the Node ID of Table 4. An example of this request is illustrated in Listing 2.

This API is the most used among QKD vendors, so end users can perform experiments as if they were directly retrieving a key from QKD equipment. Again, as the key material forwarding and network topology are managed internally by the software stack, three virtual QKD links are exposed to the end user, the ones corresponding to the two physical links and one virtual end-to-end link.

**Listing 2.** ETSI GS 014 enc_keys and dec_keys example from CCS to CAIT.

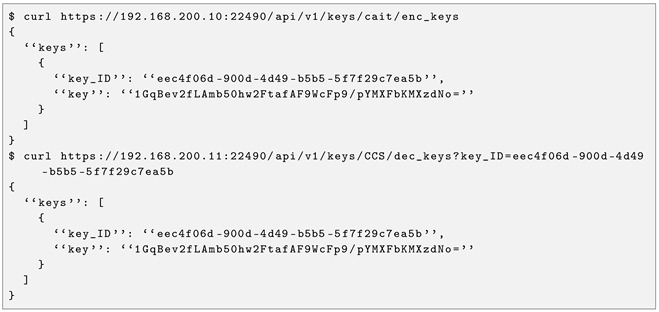



#### SD-QKD Stack: ETSI GS QKD 004

The SD-QKD software stack also exposes the ETSI GS QKD 004 interface, which fits better with the session concept and the SDN programmability of quantum network resources.

Since this standard is open to development, a REST API with three operations was implemented to expose the three 004 functions. The open_connect function, accessible through the URL /api/open_connect, is implemented as a POST request (see Listing 3 for an example of request and response). The get_key function, accessible through /api/get_key, is implemented as a GET request (see Listing 4 for an example of request and response). Finally, the close function, accessible via /api/close, is implemented as a POST request.

This interface is quality of service-driven, so that the end user can program the network to fulfil the submitted requirements: maximum and minimum key rate, key chunk size, etc. In addition to the advantages outlined in the previous sections, this interface allows multiple quantum-enabled services to be built based on the different possibilities of the API. For example:

- A general-purpose IaaS can be delivered, exposing to the end user a functionality that allocates and deallocates key material streams on demand, to experiment on QKD virtualisation and deployment, for example.
- A purpose-specific PaaS can be delivered to the user by providing a software development kit that supplies the end user with cryptographic keys in pre-established formats: for AES-128 with rekeying each second, for single-use HMAC-256, for one-type-pad stream ciphers, etc.
- A SaaS text interface can be integrated into the virtual machine to provide a fixed-length distributed seed that gives resilience to a testing gambling backend.

Thus, several quantum-enabled services can be built on top of this one in a cloud manner.

**Listing 3.** ETSI GS 004 open_connect example from CCS to CAIT.

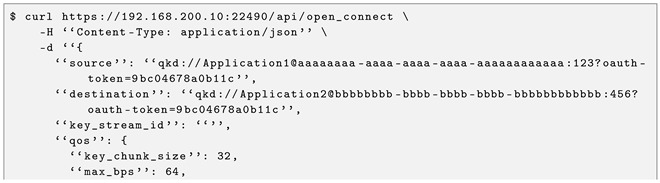



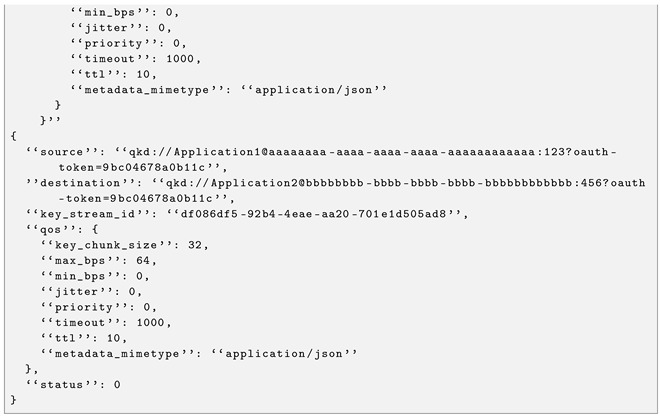



**Listing 4.** ETSI GS 004 get_key example from CCS to CAIT.

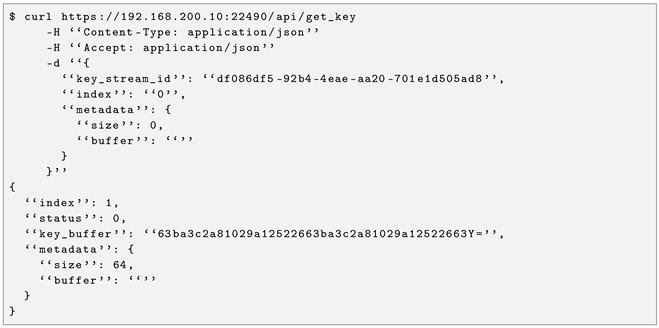



### 4.3. QRNG as a Kernel Module

To expose the QRNG, a QuSide Garnet™ was procured and embedded in the Rectorado server—the CCS server had its two long-profile PCIe slots occupied by network interface cards that provided connectivity to the two end nodes, CAIT and Rectorado.

A Linux kernel module was developed to expose its functionality to the user space, enabling the integration of an external Quside Garnet™ as an entropy source. The module registers a /dev/qrng device and associates the read() system call with a custom handler—shown in Listing 5—which is invoked whenever a user-space process requests data from the device. Upon a read operation, the handler retrieves a 32-byte Base64-encoded key from Quside Garnet™ QRNG through a secure communication interface and returns the obtained random bytes to the caller. This design allows transparent consumption of quantum-generated entropy using standard file I/O semantics while preserving kernel–user space isolation and enabling seamless integration with existing applications that rely on character devices for randomness acquisition.

**Listing 5.** Read operation to the developed QRNG kernel module.

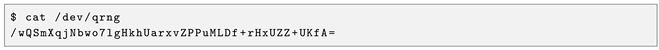



In this case, anything as a service can be built on top of this feature by invoking the /dev/qrng inside the virtual machine served. However, despite the power delivered to the end user, this feature does not allow services to be delivered in a cloud-based manner, as it is not possible to serve a large number of users. These types of features, as is the case with GPU cards, for example, can usually be enabled through software licensed by the manufacturer, which allows delivery to multiple virtual machines.

### 4.4. QRNG: OpenAPI

To address the issue discussed in the previous section, the capabilities of the same QRNG were exposed using an OpenAPI [33] interface.

Thus, to retrieve a 32-byte Base64-encoded QRNG key from the QuSide Garnet™, a request such as the one shown in Listing 6 must be issued.

**Listing 6.** OpenAPI request to retrieve entropy bytes.

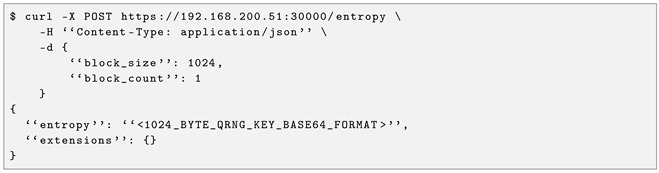



In this case, the discussion is similar to that in Section 4.2 for ETSI GS QKD 004. An API such as the one provided enables the building of quantum-enabled services at multiple levels and for both general and specific purposes.

### 4.5. TLS

TLS provides a secure, negotiated, and tunnelled connectivity service that can be protected by QKD. In this case, the TLS implementation described in [34] was used to enable on-demand quantum-enabled TLS services to deliver quantum-XaaS.

An example for deploying a TLS session from CAIT to Rectorado nodes, with key forwarding in CCS, is shown in Listing 7.

**Listing 7.** CURL HTTP GET request to deploy a TLS session between CAIT and Rectorado.

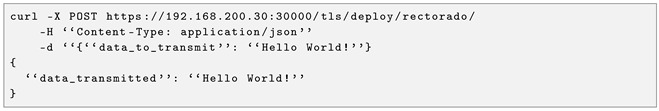



This implementation allows two sockets to be associated using the TLS implementation described above for the experiment conducted by the end user. It therefore requires expert handling of this type of connectivity but is suitable for many of the usual quantum cryptography demos. Research is being conducted on how to deliver this service to the user—e.g., by providing the user with the socket descriptor—as well as how to build quantum-XaaS services with this enabling technology.

### 4.6. IPsec

Analogously, a REST API to expose quantum-enabled IPsec services has been implemented. This API enables the deployment of IPsec tunnels in VPN mode between any link of the scenario using the implementation described in [35].

In this approach, a server machine was created in CCS, CAIT, and Rectorado nodes. This server is in charge of deploying the quantum-resistant IPsec tunnel when it receives a request from the developed REST API (see Listing 8 for an example).

**Listing 8.** CURL HTTP GET request to deploy an IPsec tunnel between CAIT and Rectorado.

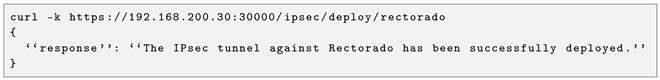



**Figure 9:**
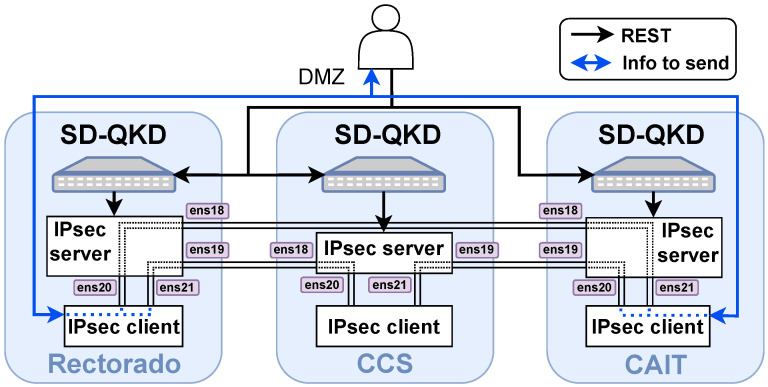
IPsec scheme overview as implemented to serve IPsec-based quantum-XaaS.

The overall approach is illustrated in Figure 9. Each node hosts an IPsec server responsible for establishing IPsec tunnels with other nodes using quantum-distributed keys retrieved from the SD-QKD server. In addition, an IPsec client is deployed within each node, to which external users are granted access. This approach was originally based on the trusted node model, which is why an agent-based approach was chosen, among other advantages.

Each IPsec server contains four network interfaces: two dedicated to inter-node communication with the other IPsec servers, and two used for intra-node communication with the local IPsec client. These interfaces are statically configured, as shown in Figure 9. Once an external user accesses the IPsec client, an IPsec tunnel deployment can be requested. Upon receiving a request such as the one shown in Listing 8, an IPsec tunnel is established between the CAIT IPsec server and the Rectorado IPsec server, and the corresponding firewalls route all traffic through the appropriate interfaces. The end user then only needs to send traffic through the appropriate interface of the IPsec client to communicate with the remote endpoint.

When the end user wishes to terminate the IPsec tunnel, a destroy request must be issued, such as the one shown in Listing 9.

**Listing 9.** CURL HTTP POST request to remove the IPsec tunnel between CAIT
and Rectorado.

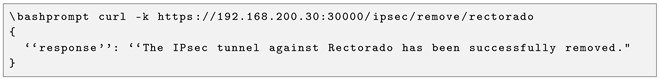



The discussion of this solution is very similar to that of TLS-based services in the previous section. It requires expert handling of this type of technology, but IPsec is fundamental in many of the demonstrations made with QKD technology in the current landscape. It is also the technology underlying VPNs, making it possible to experiment with quantum-protected VPN-aaS service delivery models.

### 4.7. Applicability in Cloud Environments

The applicability of the solutions presented in this work relies on the principles outlined in Section 2: hardware abstraction, multi-tenancy, access through standardised APIs, and elasticity in provisioning. In this sense, these solutions were conceived to satisfy these principles and therefore act as a foundation upon which to build quantum-enabled services directly applicable to cloud environments.

The quantum-enabled services described are enabling technologies for cloud environments as they abstract resources tied to the quantum network hardware—i.e., key streams, quantum entropy, and secure tunnels—into API-accessible interfaces. They provide on-demand, programmable provisioning of quantum-distributed keys, quantum-generated entropy, or quantum-secured tunnels while also enabling multi-tenancy through the virtualisation and logical partitioning of the resources. Moreover, each quantum-enabled service can support others; low-level capabilities, such as the quantum-distributed key, can enable intermediate services, such as quantum-secured tunnels, which, in turn, support higher-level applications.

The adaptability of the proposed solutions to cloud infrastructures and technologies is ensured by the use of standards and REST APIs, which facilitate integration with existing platforms. Indeed, the SD-QKD stack exposes the ETSI GS QKD 004 and 014 interfaces and operates according to the ETSI GS QKD 015 workflows. A similar approach is used to expose entropy via an OpenAPI interface and to drive IPsec and TLS deployments through simple REST calls. However, it is important to be aware that adaptability also lies in how these services are exposed to provide particular services and support higher-level ones. The solutions explained in this work are therefore useful building blocks for adapting quantum network systems to cloud environments, but new features need to be developed on top of them.

A similar discussion is possible regarding scalability. The QKD systems integrated in the infrastructure—HEQA for short spans and Toshiba for longer ones—deliver sufficient key material to support multiple concurrent end users. In addition, the use of the SD-QKD software stack, based on software-defined networking techniques and scalable quantum network resource management [1], enables several tenants to operate on the same physical links without replicating hardware. Finally, the scalability of the rest of the quantum-enabled services proposed in this work is ensured as they are based on well-known techniques and technologies, e.g., TLS and IPsec.

Thus, the proposed approach demonstrates that quantum network capabilities can be exposed and operated according to cloud principles while remaining compatible with existing infrastructures. The standards-driven SD-QKD workflows and simple REST interfaces for QRNG, TLS, and IPsec result in building blocks directly usable in cloud environments and able to support higher-level services.

## 5. Conclusions and Future Work

The MadQCI ecosystem, in alignment with EuroQCI quantum network deployments, investigates how quantum-enabled services can be delivered to end users. In this work, the foundations for this functionality using frameworks and techniques related to cloud environments are set.

The proposed framework is based on the anything-as-a-service (XaaS) approach, the viability of which for delivering quantum-enabled services has been discussed. Included is its applicability to both general- and specific-purpose applications, as well as its viability in terms of adaptability and scalability.

To this end, a dedicated quantum network scenario was deployed, consisting of IT systems distributed across up to three different remote locations within the MadQCI ecosystem to establish a data centre-like infrastructure. Two QKD systems and one QRNG were specifically chosen for this application and integrated to provide quantum communication capabilities to the cloud environment. These capabilities were quantified using real data such as their cost.

Finally, several software developments that provide quantum-enabled services through cloud-style services to potential end users have been reported in this work. These implementations have already leveraged quantum communication technologies—an SD-QKD stack and its ETSI GS QKD 004 and ETSI GS QKD 014, TLS, and IPsec interfaces—but have been fitted with interfaces, technologies, and capabilities tailored to the cloud environment. This approach enables the creation of new XaaS services or, as detailed, quantum-IaaS, -PaaS, -SaaS, or -FaaS services.

### Future Work

This work presents a preliminary effort, the full potential of which has yet to be evaluated. The performance of the proposed frameworks and solutions will need to be assessed with real end users once the described scenario has been in use for several months. In this regard, it will be essential to evaluate how the approach adapts and scales to the user demands in order to verify that the proposed solutions enable the delivery of quantum-enabled services in a ubiquitous, user-friendly, on-demand, and elastic manner consistent with a cloud-based approach.

## Figures and Tables

**Table 1:** Analysis of the MadQCI cloud scenario using the same approach as [16].

Cloud Characteristic	Assessment
Appearance of infinite computing resources on demand	Both quantum network systems and quantum-enabled services are delivered in an abstract manner to the users, e.g., using ETSI ISG QKD standard on-demand APIs.
Elimination of an up-front commitment by cloud users	The services are delivered in an open and collaborative manner within the context of R&D projects, without requiring any initial investment or specific prerequisites.
Ability to pay for computing resources on a short-term, on-demand basis	The provided services are easily segmented. For example, the use of the ETSI IGS QKD standard APIs can be billed according to the amount of key material extracted, while other infrastructure services can be charged on a time basis.
Economies of scale due to very large data centres	In the context of quantum communications R&D, systems are very expensive—e.g., a single QKD pair costs around €150,000; this scenario would be impossible without a MadQCI ecosystem outfitted with 23 QKD systems.
Higher utilisation through the multiplexing of workloads from different organisations	QKD or QRGN systems are not used continuously and this form of opening MadQCI will maximise their use and prevent other researchers from procuring systems.
Simplify operation and increase utilisation via resource virtualisation	The SD-QKD stack manages multi-tenancy in a scalable manner by handling the quantum-distributed keys as virtual streams of key material.

**Table 2:** Proposed approach for quantum-enabled cloud-type services.

Cloud Approach	Service Exposed
quantum-IaaS	Virtualised quantum infrastructure methods and systems to support quantum-enabled services.
quantum-PaaS	Abstract and complete set of quantum-enabled methods to build new quantum-enabled services on top.
quantum-SaaS quantum-FaaS	Quantum-enabled, ready-to-run methods that solve any general-purpose or specific-purpose problems.

**Table 3:** CAPEX cost of the potential TLS services delivered, as shown in Figure 7, assuming a depreciation period of 5 years and a 360-day calendar, as well as ignoring the cost of the optical connectivity.

Service	Cost [€]
Intra-data centre TLS AES 128 GCM SHA 256 using a HEQA QKD system	0.000014
Inter-data centre TLS AES 128 GCM SHA 256 using a HEQA QKD system	0.000019
Intra-data centre TLS AES 256 GCM SHA 384 using a HEQA QKD system	0.000019
Inter-data centre TLS AES 256 GCM SHA 384 using a HEQA QKD system	0.000028
Intra-data centre TLS AES 128 GCM SHA 256 using a Toshiba QKD system	0.00000042
Inter-data centre TLS AES 128 GCM SHA 256 using a Toshiba QKD system	0.00000107
Intra-data centre TLS AES 256 GCM SHA 384 using a Toshiba QKD system	0.00000059
Inter-data centre TLS AES 256 GCM SHA 384 using a Toshiba QKD system	0.00000138

**Table 4:** Legend for retrieving a QKD key between SD-QKD nodes.

Location	Node ID	Hostname
Rectorado	rectorado	192.168.200.30:30000
CCS	CCS	192.168.200.10:30000
CAIT	cait	192.168.200.50:30000

## Data Availability

Data will be made available upon reasonable request.

## Abbreviations

The following abbreviations are used in this manuscript:

API	Application Programmable Interface
AES	Advanced Encryption Standard
CCS	Center for Computational Simulation
CEM	Centro Español de Metrología
CETSE	Centro Tecnológico de Seguridad
CSIC	Consejo Superior de Investigaciones Científicas
CURL	Client Uniform Resource Locator
ETSI	European Telecommunications Standards Institute
FaaS	Function as a Service
HMAC	Hash-Based Message Authentication Code
HTTP	Hypertext Transfer Protocol
IaaS	Infrastructure as a Service
IMDEA	Instituto Madrileño de Estudios Avanzados
INTA	Instituto Nacional de Técnicas Espaciales
IPsec	Internet Protocol Security
MadQCI	Madrid Quantum Communications Infrastructure
PCIe	Peripheral Component Interconnect Express
PQC	Post-Quantum Cryptography
QBER	Quantum Bit Error Rate
QKD	Quantum Key Distribution
QRNG	Quantum Random Number Generator
SaaS	Software as a Service
RBAC	Role-Based Access Control
RM	REDIMadrid
REST	Representational State Transfer
SDN	Software-Defined Networking
SD-QKD	Software-Defined Quantum Key Distribution
TInD	Telefónica Innovación Digital
TLS	Transport Layer Security
UPM	Universidad Politécnica de Madrid
URL	Uniform Resource Locator
VPN	Virtual Private Network

## References

[1] Martin V., Brito J.P., Ortiz L., Mendez R.B., Buruaga J.S., Vicente R.J., Sebastián-Lombraña A., Rincon D., Perez F., Sanchez C. (2024). MadQCI: A heterogeneous and scalable SDN-QKD network deployed in production facilities. NPJ Quantum Inf..

[2] Martin V., Martinez-Mateo J., Peev M., Webster J.G. (2017). Introduction to Quantum Key Distribution. Wiley Encyclopedia of Electrical and Electronics Engineering.

[3] Gisin N., Ribordy G., Tittel W., Zbinden H. (2002). Quantum cryptography. Rev. Mod. Phys..

[4] (2024). The European Quantum Communication Infrastructure (EuroQCI) Initiative.

[5] Sebastián-Lombraña A., Rincón D., Brito J.P., Ortiz L., Martín V. (2024). A purpose-specific design for the next iteration of the Madrid quantum network. Proceedings of the 2024 24th International Conference on Transparent Optical Networks (ICTON).

[6] Buruaga J.S., Fernández D., Sebastián-Lombraña A., Vicente R.J., Brito J.P., Ortiz L., Rosales J.L., Martín V. (2025). Opening MadQCI: A quantum-enabled time-aware distributed data centre for open R&D. Proceedings of the 2025 25th Anniversary International Conference on Transparent Optical Networks (ICTON).

[7] (2020). Quantum Key Distribution (QKD); Application Interface.

[8] (2019). Quantum Key Distribution (QKD); Protocol and Data Format of REST-Based Key Delivery API.

[9] (2024). OpenQKD Project Website. https://openqkd.eu/.

[10] MacQuarrie E.R., Simon C., Simmons S., Maine E. (2020). The emerging commercial landscape of quantum computing. Nat. Rev. Phys..

[11] Aleksandrowicz G., Alexander T., Barkoutsos P., Bello L., Ben-Haim Y., Bucher D., Jose Cabrera-Hernández F., Carballo-Franquis J., Chen A., Chen C.F. (2019). Qiskit.

[12] Chou H.P., Hongfu C. (2023). QRNG Entropy as a Service(EaaS) Platform for Quantum-Safe Entropy and key Delivery.

[13] Silva N.A., Ferreira M.J., Carvalho A., Souto A., Paunković N., Mateus P., Teixeira A., Pinto A.N. (2023). A Network Server for Distributing Quantum Random Numbers. Proceedings of the 2023 23rd International Conference on Transparent Optical Networks (ICTON).

[14] Martin R., Lopez B., Vidal I., Valera F., Nogales B. (2024). Service for Deploying Digital Twins of QKD Networks. Appl. Sci..

[15] Vendrell A., Moreno J. (2024). Monitoring a Quantum Network on AWS. AWS Quantum Technologies Blog. Contributed by LuxQuanta and AWS, Describing Integration of LuxQuanta’s NOVA LQ CV-QKD System with AWS Infrastructure for Centralized Telemetry. https://aws.amazon.com/cn/blogs/quantum-computing/monitoring-a-quantum-network-on-aws/.

[16] Armbrust M., Fox A., Griffith R., Joseph A.D., Katz R., Konwinski A., Lee G., Patterson D., Rabkin A., Stoica I. (2009). Above the Clouds: A Berkeley View of Cloud Computing.

[17] Mell P., Grance T. (2011). The NIST Definition of Cloud Computing.

[18] (2014). Information Technology—Cloud Computing—Overview and Vocabulary.

[19] Cao Y., Zhao Y., Wang Q., Zhang J., Ng S.X., Hanzo L. (2022). The Evolution of Quantum Key Distribution Networks: On the Road to the Qinternet. IEEE Commun. Surv. Tutor..

[20] Renner R., Gisin N., Kraus B. (2005). Information-theoretic security proof for quantum-key-distribution protocols. Phys. Rev. A.

[21] Mavroeidis V., Vishi K., Zych M.D., Jøsang A. (2018). The Impact of Quantum Computing on Present Cryptography. Int. J. Adv. Comput. Sci. Appl..

[22] Singh R., Hill C., Kawaguchi S., Lupo J. Secure Key Integration Protocol (SKIP). Internet-Draft Draft-Cisco-Skip-02, Internet Engineering Task Force, 2025. Work in Progress. Expires March 7, 2026. https://datatracker.ietf.org/doc/draft-cisco-skip/02/.

[23] National Institute of Standards and Technology (2020). Recommendation for Key Management: Part 1—General.

[24] Vernam G.S. (1926). Cipher Printing Telegraph Systems For Secret Wire and Radio Telegraphic Communications. Trans. Am. Inst. Electr. Eng..

[25] Berrevoets R.C., Middelburg T., Vermeulen R.F.L., Chiesa L.D., Broggi F., Piciaccia S., Pluis R., Umesh P., Marques J.F., Tittel W. (2022). Deployed measurement-device independent quantum key distribution and Bell-state measurements coexisting with standard internet data and networking equipment. Commun. Phys..

[26] Bernstein D.J., Buchmann J., Dahmen E. (2009). Post-Quantum Cryptography.

[27] Chen L., Jordan S., Liu Y.K., Moody D., Peralta R., Perlner R., Smith-Tone D. (2016). Report on Post-Quantum Cryptography.

[28] OpenSSL Software Foundation (2025). OpenSSL 3.5 Final Release. https://openssl-library.org/post/2025-04-08-openssl-35-final-release/.

[29] Stebila D., Fluhrer S., Gueron S. Hybrid Key Exchange in TLS 1.3. Internet-Draft Draft-Ietf-Tls-Hybrid-Design-16, Internet Engineering Task Force, 2025. Work in Progress. https://datatracker.ietf.org/doc/draft-ietf-tls-hybrid-design/.

[30] ETSI (2020). Quantum Key Distribution (QKD); Application Interface.

[31] ETSI (2019). Quantum Key Distribution (QKD); Protocol and data format of REST-based key delivery API.

[32] ETSI (2022). Quantum Key Distribution (QKD); Control Interface for Software Defined Networks.

[33] Palo Alto Networks (2025). QRNG Open API. GitHub Repository. https://github.com/PaloAltoNetworks/QRNG-OPENAPI/blob/main/GitHub%20Repository%20for%20QRNG%20Open%20API.md.

[34] Buruaga J.S., Méndez R.B., Brito J.P., Martin V. (2025). Hybrid Quantum-Safe integration of TLS in SDN networks. Comput. Netw..

[35] Brito Mendez R.D., Buruaga J.S., Brito J.P., Pastor A., Lopez D.R., Martin V. (2025). Quantum Resistant Software Defined-Networking IPsec: Enabling its Communication Over IP Networks on Real Telco Infrastructures. SSRN Electron. J..

